# Weight-band-based simplification of oral allometric miltefosine dosing in paediatric patients with visceral leishmaniasis

**DOI:** 10.1093/jac/dkag014

**Published:** 2026-01-23

**Authors:** Andres Mazariegos Herrera, Mats O Karlsson, Elin M Svensson, Thomas P C Dorlo

**Affiliations:** Department of Pharmacy, Uppsala University, Uppsala, Sweden; Department of Pharmacy, Uppsala University, Uppsala, Sweden; Department of Pharmacy, Uppsala University, Uppsala, Sweden; Department of Pharmacy, Pharmacology and Toxicology, Radboud University Medical Center, Nijmegen, The Netherlands; Department of Pharmacy, Uppsala University, Uppsala, Sweden

## Abstract

**Background and objectives:**

An allometric miltefosine regimen dosed based on fat-free mass (FFM) is effective and safe in treating children with visceral leishmaniasis (VL). However, its complexity hinders successful implementation in endemic areas. We aimed to develop a simplified dosing based on weight bands (WBs) that achieves equivalent miltefosine exposure in a paediatric VL population using a simulation-based approach.

**Methods:**

Utilizing demographic data from 9379 Eastern African paediatric VL patients, WHO–CDC growth curves were adjusted to create a realistic virtual paediatric VL population. The virtual children were given either an allometric FFM-based, a WB-based or a 2.5 mg/kg dosing of miltefosine per day. To compare the regimens, two pharmacokinetic metrics were derived from the simulated patient population receiving the allometric FFM-based regimen: the 5th percentile of the time above the concentration associated with 90% *in vitro* intracellular parasite killing for efficacy and the 95th percentile of the AUC for safety. The performance of the dosing regimens was evaluated for both 14- and 28-day regimens.

**Results:**

A virtual population was constructed that closely resembled real-world paediatric Eastern African VL patients’ height- and weight-for-age distributions. Target attainment rates for the two pharmacokinetic metrics tested differed by less than 1.5% between the final WB- and FFM-based dosing regimens. The final doses in mg were 20 for children under 6 kg, 30 for 6.0–9.9 kg, 50 for 10.0–14.9 kg, 60 for 15.0–19.9 kg, 70 for 20.0–24.9 kg and 80 for 25.0–29.9 kg, for both 14- and 28-day regimens.

**Conclusions:**

Our simplified WB-based dosing strategy offers a practical alternative for allometric miltefosine dosing in children, yielding satisfactory exposure levels.

## Introduction

Visceral leishmaniasis (VL) is a neglected tropical disease caused by the protozoan *Leishmania* parasites.^1^ The main clinical symptoms are hepatosplenomegaly and prolonged fever.^2,3^ The disease is considered fatal if left untreated.^4^ Although the disease is endemic in over 70 countries worldwide,^1^ the majority of current cases occur in the Eastern African region, with over 50% of incidences in children under 15 years old.^5,6^ It is imperative to provide accessible, effective and safe antileishmanial drug regimens specifically adapted to the paediatric population in need.^7,8^

Dosing of miltefosine, the only existing oral drug for the treatment of leishmaniasis, was initially linearly extrapolated from the recommended dose in adults based on body weight, i.e. 2.5 mg/kg/day for 28 days.^9–11^ Clinical pharmacokinetic (PK) studies on miltefosine in both South Asia and Eastern Africa found that this mg/kg dosing in children with leishmaniasis led to markedly lower miltefosine exposure compared with adults.^12–15^ Moreover, low exposure to miltefosine was significantly associated with a higher risk of relapse and treatment failure at 6-month follow-up^14^ and has been associated with low efficacy rates of 59% and 65% in Eastern African and Nepalese paediatric VL patients, respectively.^15,16^ The time that the miltefosine plasma concentration was above the *in vitro* intracellular *Leishmania donovani* susceptibility value (10.6 µg/mL) associated with 90% intracellular parasite killing (T > EC_90_) was identified as a relevant PK metric associated with a reduction in the risk of infection relapse during a 6-month follow-up period.^13^

To overcome the discrepancy in exposure between paediatric and adult VL patients, a dosing regimen for children below 30 kg using the body size descriptor fat-free mass (FFM) was proposed.^17^ FFM is derived from the paediatric patients’ sex, height and weight.^18,19^ This dosing regimen translates into higher mg/kg doses for children and was clinically evaluated for safety, efficacy and PK in Ugandan and Kenyan paediatric VL patients. Results indicated a similar exposure compared with adults on 2.5 mg/kg/day miltefosine, without an increase in adverse effects.^16^ In terms of efficacy, the FFM-based regimen achieved a 90% cure rate, comparable to the cure rate in adults on the mg/kg dosing.^16,20^ A follow-up Phase 3 study looked into shortening the treatment duration from 28 to 14 days when administered with paromomycin, with comparable miltefosine levels between children and adults at the end of treatment. The results demonstrated that 14-day combination therapy was similar in efficacy to the standard sodium stibogluconate–paromomycin combination, the current standard of care for VL treatment in Africa.^21,22^

The allometric FFM-based regimen has over 50 different dosing categories based on the patient’s weight, height and sex (Table S1, available as Supplementary data at *JAC* Online), potentially causing prescription errors. Establishing the correct dose could be challenging in the communities most affected by VL, potentially leading to adherence issues.^23,24^ A simpler paediatric dosing approach is to use weight bands (WBs). A project fostered by the WHO Global Accelerator for Pediatric Formulations (GAP-f) has recently proposed harmonized WB ranges to dose children across various infectious diseases. This effort aims to avoid dosing errors and encourage the use of WB-based dosing in future paediatric drug development.^25^ In the current study, we aimed to simplify the allometric FFM-based miltefosine dosing regimen into harmonized WBs while ensuring equivalent drug exposure. A final, unified dose recommendation for both 14- and 28-day miltefosine regimens using the proposed harmonized WBs was determined.

## Methods

### Virtual paediatric Eastern African VL population

Demographic data for 9379 VL paediatric patients (age range 0–18 years) were obtained from various sources, including observational data from Médecins Sans Frontières (MSF; South Sudan and Ethiopia) and clinical trial and pharmacovigilance trial data from the Drugs for Neglected Diseases initiative (DNDi; Ethiopia, Kenya, Sudan and Uganda). Data included age, weight, height and country of origin of patients from South Sudan (*n* = 6396), Sudan (*n* = 1371), Ethiopia (*n* = 660), Kenya (*n* = 782) and Uganda (*n* = 170). A correlation factor of weight and height was established through the individual z-scores for height-for-age and weight-for-age for each sex independently.

We built a virtual population using growth charts from the WHO and the CDC, which are reference growth curves commonly used to simulate weight and height by sex and age of healthy children. They contain weight- and height-for-age LMS values, where L is the exponent for the Box–Cox transformation (representing skewness), M is the median and S is the coefficient of variation.^26,27^ The WHO weight-for-age curves are only available until an age of 10 years, so the corresponding weight-for-age CDC curves from 10 years and above were merged to ensure continuous curves up until 18 years old.^28^ Using the approach described by Wasmann *et al.*,^28^ the correction of the z-score for weight- and height-for-age from real-world patients was used to generate 300 individuals for each age-month.

In short, the virtual WHO–CDC multivariate population demographic distributions were matched to the collected real-world data from paediatric VL patients using a polynomial correction function that scaled the height- and weight-for-age distributions per sex.

### Population PK models for simulation

A recently published population PK model developed by Verrest *et al*.^21^ was used to simulate individual concentration–time profiles of miltefosine. The model was based on data from a Phase 3 study conducted in Kenya, Sudan, Ethiopia and Uganda in adults and children (≥4 years of age) with VL receiving either 14 or 28 days of allometric FFM-based miltefosine doses in combination with paromomycin. It consists of a two-compartment distribution model with first-order absorption and elimination and two covariate effects associated with a decrease in bioavailability. Additionally, the clearance and volume of distribution of the central compartment were allometrically scaled with FFM. PK parameter estimates used for the simulation can be found in Table S2. Monte Carlo simulations were conducted including inter-individual variabilities (IIVs).

### Evaluated dosing regimens

Three miltefosine dosing regimens were simulated and compared: (i) the *mg/kg dose* of 2.5 mg/kg/day, (ii) the allometric *FFM-based dose* and (3) the allometric *WB-based dose.* For the 2.5 mg/kg/day regimen, patients’ doses were rounded to the nearest 10 mg. The FFM-based regimen proposed by Dorlo *et al*.^17^ allometrically scales clearance using FFM as a body size descriptor to achieve comparable miltefosine exposures in children and adults. The FFM-based dose scales the standard dose of 150 mg (Dose_std,adult_) for a reference adult FFM of 53 kg (FFM_ref,adult_) with the individual paediatric FFM (FFM_i_) using a fixed power exponent of 0.75, as seen in Equation 1. FFM_i_ was estimated using the equations by Janmahasatian *et al*.^18^


(1) $$Dose_{FFM}=Dose_{std,adult}\times {\left(\frac{FFM_{i}}{FFM_{std,adult}}\right)}^{0.75}$$

Miltefosine is available as 10 and 50 mg capsules, meaning that a 90 mg dose would require five capsules and a mix of both capsule strengths. To reduce pill burden, previous clinical studies rounded the dose to 100 mg (two capsules) instead, and this dose was also used in our simulations.^22^ For the WB-based dosing, the WB ranges harmonized across therapeutic areas recently proposed by Waalewijn *et al.*^25^ were applied (Table 1).

**Table 1.** Summary of selected doses per WB for the allometric WB-based regimen

	Score-based selected doses		
Weight band range (kg)	14-day WB-based (mg)	28-day WB-based (mg)	Final unified daily dose (mg)
<6	20	20	20
6.00–9.99	30	30	30
10.00–14.99	40	50	50
15.00–19.99	60	60	60
20.00–24.99	70	70	70
25.00–29.99	80	80	80

kg, kilogram; mg, milligram; WB, weight band.

### Target exposure metrics

To compare the regimens, two target exposure metrics were derived based on the allometric FFM-based dose from start till end of treatment (EOT): as a lower exposure target, the 5th percentiles of the T > EC_90,0-EOT_, reflecting its reported association with reduced relapse risk in previous studies.^14^ Since no exposure–toxicity relationships have been established for miltefosine, the 95th percentiles of AUC_0-EOT_ were used as the upper exposure target. Separate lower and upper exposure targets were proposed for 14- and 28-day regimens.

### Dose selection

A score (Score_WB_) was used to determine the optimal dose for each WB, which considered the upper and lower target achievement with equal weight. Score_WB_ was based on the number of patients outside the targets within each WB as described in Equation 2. For each WB, the sum of individual patients that are over the upper (n_upper_) and under the lower (n_lower_) targets was scaled by the total number of simulated individuals within each WB (n_total_). The dose selection per WB was based on the lowest Score_WB_. The final score can also be interpreted as the sum of all percentages of patients outside the upper and lower targets.


(2) $$Score_{WB}=\;\underset{WB}{\sum }\frac{\;n_{upper}+\;n_{lower}}{n_{total}}$$

Dose selection was performed for both 14- and 28-day treatments. The aim was to propose a unified WB-based dosing regimen applicable to both treatment durations. When different doses were recommended for the same WB, the highest dose was selected to maximize exposure (T > EC_90,0-EOT_),^14^ and exposure levels were compared with reported concentrations in a sensitivity analysis.^16,22^

### Software

Data management, preprocessing, polynomial fitting, creation of the virtual population and graphical analysis were carried out in R (v. 4.5.1) using Rstudio (v. 2025.09.0+387) as interface. Individual PK profiles were simulated using the *mrgsolve* package in R (v. 1.5.2).^29^

## Results

### Virtual paediatric Eastern African VL population

**Figure 1.**
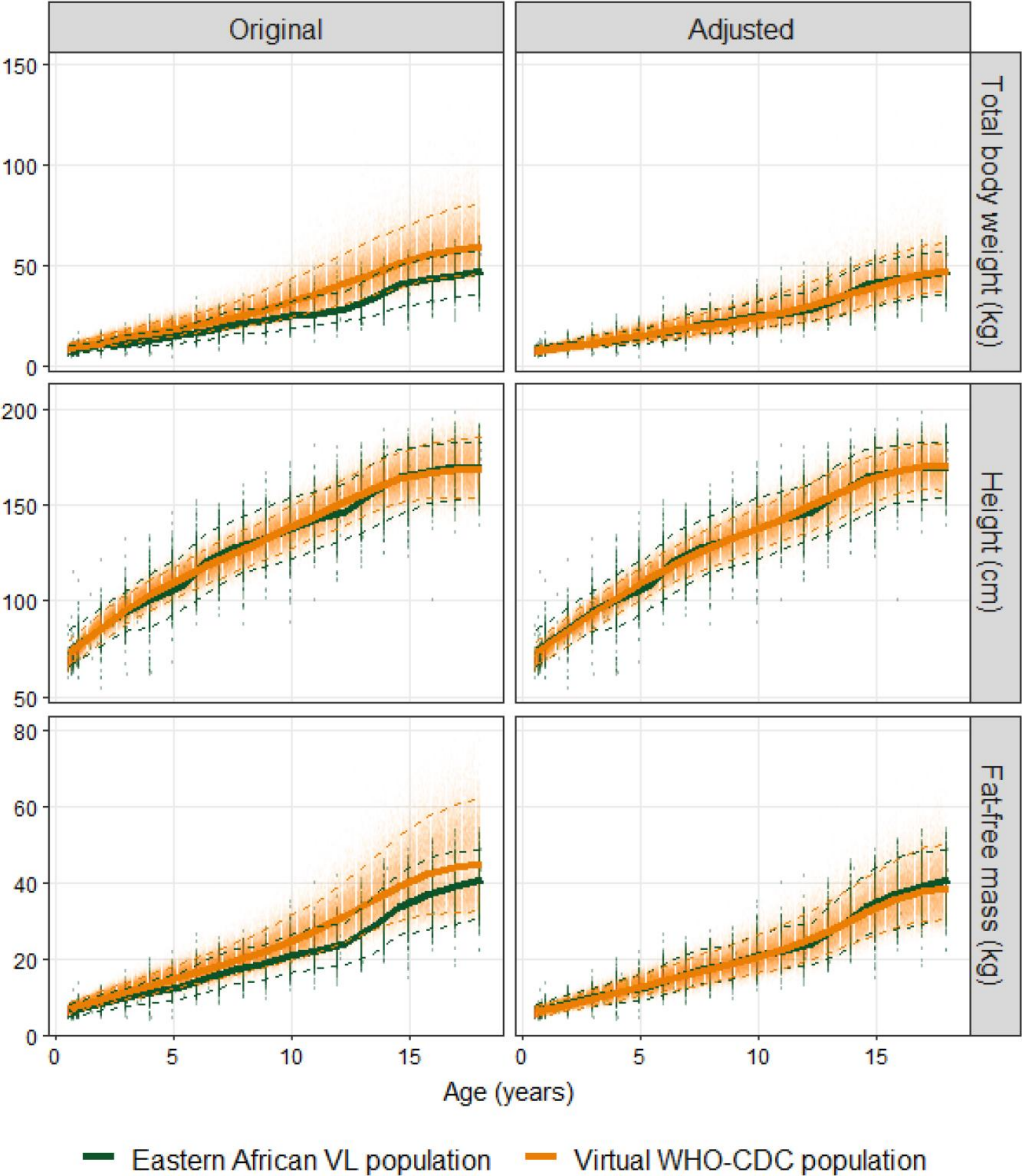
Growth curves in terms of total body weight- (upper panels), height- (mid-panels) and FFM-for-age (lower panels) showing the observed and simulated distributions before the inclusion of VL-specific correction factors (left column) and after (right column). Points represent observed and simulated data, while lines represent the median (solid), 5th and 95th percentiles (dashed) of the respective groups.

Eastern African paediatric VL patient data showed pronounced differences compared with the standard WHO–CDC growth curves (Figure 1). Both weight- and FFM-for-age of VL patients were lower (−14 to −32%) than their WHO–CDC-generated counterparts. When stratifying on sex, height-for-age curves indicated minor differences for children over 12 years of age, with girls being taller than the median WHO–CDC curve, while boys are shorter (Figure S1). After scaling the heights and weights with polynomial correction functions (Table S3), the observed and simulated medians and distribution for weight-, height- and FFM-for-age of the adjusted WHO–CDC virtual population and the Eastern African VL population resulted in a good agreement (Figure 1).

### Target exposure metrics and dose selection

The lower exposure targets (T > EC_90,0-EOT_) were 3 and 17 days, while the upper (AUC_0-EOT_) were 262 and 740 µg *day/mL for the 14- and 28-day treatment duration, respectively. Based on these derived targets, the selected doses for the WB-based regimen were the same for both treatment durations across all WBs except for the 10.00–14.99 kg interval, where the daily dose for 14-day treatment was 40 and 50 mg for the 28-day treatment (Table 1). This dose was set to 50 mg to have a unified dose recommendation.

### Target attainment

**Figure 2.**
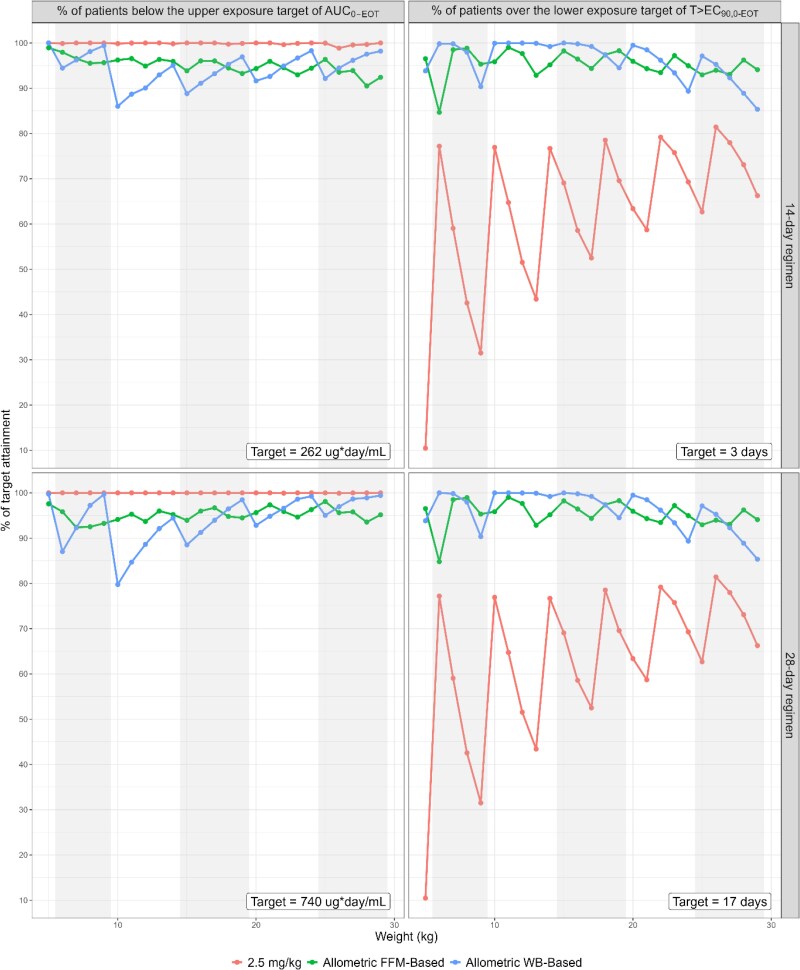
Target attainment for the various dosing regimens in percentages, stratified by treatment duration of either 14 days (top) or 28 days (bottom), and by exposure target with the lower exposure target T > EC_90,0-EOT_ (left) and the upper exposure target AUC_0-EOT_ (right) with the respective target values in the bottom right corner. Shading indicates the separate WBs.

For both treatment durations, 85%–100% of patients in each kg weight were over the lower T > EC_90,0-EOT_ target with the allometric FFM- and WB-based regimens, while the mg/kg dosing regimen exhibited a substantially lower target attainment between 10% and 81% (Figure 2). The lowest attainment of the T > EC_90,0-EOT_ target for the WB-based regimen occurred in the highest-weight patients within each WB (as low as 85%), while the lowest upper target attainment (as low as 80%) was seen in the lowest-weight patients within each WB.

The difference in total score of the WB-based and the FFM-based regimens was relatively small (Table 2), with only small deviations in the separate target attainments. Whereas the FFM-based regimen had a lower proportion of patients over the upper exposure target, the WB-based regimen had a lower proportion of target attainment for the lower exposure target in both treatment durations. In contrast, the 2.5 mg/kg dosing had the worst performance of target attainment, with up to 36% being below the lower target, indicating insufficient miltefosine exposure.

**Table 2.** Summary of the score for all regimens

	14-day regimen			28-day regimen		
	Below lower target^a,c^	Over upper target^b^	Total score	Below lower target^a,c^	Over upper target^b^	Total score
Allometric FFM-based	4.2	5.0	9.2	4.3	5.0	9.3
Allometric WB-based	3.2	6.3	9.5	3.2	6.1	9.3
2.5 mg/kg	36	0.0	36	36	0.1	36

^a^The lower exposure target of T > EC_90, 0-EOT_ at 14- and 28-day regimens was 3 and 17 days, respectively.

^b^The upper exposure target of AUC_0-EOT_ at 14- and 28-day regimens was 262 and 740 µg * day/mL, respectively

^c^EC_90_ was derived from the inhibitory concentration (IC_90_) of miltefosine against the *in vitro* intra-macrophage amastigote form of the *L. donovani* parasite from Eastern African samples, with a value of 10.6 µg/mL.^14^

The distribution of various PK metrics from all simulated regimens was compared (Table 3). For most metrics, FFM-based and WB-based regimens’ performance was similar, with the latter having a slightly higher median. In contrast, the mg/kg regimen resulted in lower values across all these metrics than the two allometric-based regimens. A detailed summary by individual 1 kg weight bins is shown in Figure S2, where target attainment was generally consistent between the WB- and FFM-based regimens.

**Table 3.** Miltefosine exposure metrics (median, IQR) for the various dosing regimens

	14-day regimen			28-day regimen		
Exposure metric	Allometric FFM-based	Allometric WB-based	2.5 mg/kg	Allometric FFM-based	Allometric WB-based	2.5 mg/kg
AUC_0-EOT_ (µg * day/mL)	129 (100–176)	133 (103–183)	97.0 (75.0–132)	533 (475–606)	542 (484–617)	417 (366–476)
T > EC_90,0-EOT_ (days)	4.91 (3.92–8.03)	4.97 (3.97–8.50)	3.72 (2.77–5.65)	18.9 (17.9–22.0)	19.0 (18.0–22.5)	17.7 (16.8–19.7)
T > EC_90, 0–210d_ (days)	11.5 (9.40–15.3)	11.9 (9.73–15.7)	8.09 (6.11–11.1)	27.0 (24.4–30.2)	27.1 (24.5–30.2)	25.5 (23.1–28.5)
*C*_max, 0-EOT_ (µg/mL)	21.3 (19.4–23.6)	21.7 (19.7–24.5)	16.7 (14.9–18.9)	32.4 (29.5–35.4)	32.9 (30.1–36.0)	27.2 (24.5–30.0)

µg, micrograms; *C*_max, 0-EOT_, maximal concentration over the complete treatment period; IQR, interquartile range; kg, kilograms; mg, milligrams; mL, millilitres; T > EC_90, 0–210d_, time the miltefosine concentration is over the EC_90_ from Day 0 to Day 210 (typical follow-up time in VL studies).

## Discussion

This study presents a simplified WB-based allometric dosing regimen for oral miltefosine in paediatric patients with VL in Eastern Africa, reducing the number of dosing categories to only six while keeping exposures similar to the allometric FFM-based dosing regimen. By doing this, we aim to facilitate uptake and rollout of miltefosine treatment in remote rural areas while assuring the same exposure, safety and effectiveness as the allometric FFM-based regimen. Moreover, we aligned our WB recommendations to those promoted by the WHO GAP-f, to further allow alignment of paediatric treatment across various common concomitant diseases such as HIV, malaria and tuberculosis.^25,30–32^ Proposals to employ WB-based dosing have been recently published to optimize paediatric dosing of various tuberculosis drugs, as well as in other therapeutic areas such as malaria and HIV.^33–35^

While simplifying the highly granular protocol of FFM-based miltefosine dosing into a WB-based approach, we balanced ease of use and effectiveness. To keep the drug exposures for the WB-based regimen comparable to those achieved with the FFM-based regimen, upper and lower exposure targets were established from simulations of a realistic virtual VL Eastern African population receiving the latter. As PK metrics for these targets, we selected T > EC_90,0-EOT_ and AUC_0₋EOT_, respectively. A decrease in the risk of disease relapse has been linked to the time that the miltefosine concentration is above the *in vitro* EC_90_; hence, this was chosen as a lower exposure target. AUC_0₋EOT_ serves as an empirical upper exposure target of total exposure during treatment in the absence of established exposure–toxicity relationships.^14,36^ Together, the two metrics support a simplified dosing regimen that retains alignment with key therapeutic principles. The weight of both target metrics was assumed equal to maintain an unbiased balance between therapeutic relevance and model-based principles guiding regimen selection.

Dose selection was performed for a 14-day regimen intended as part of a combination therapy together with paromomycin and a 28-day regimen to be given as monotherapy.^22,37^ We aimed to have the same dosing recommendation for both treatment durations to further simplify the regimen despite a different dose selection between regimens for one of the six WBs. For the range of 10.00–14.99 kg, we kept the higher dose selected for the 28-day regimen, given that there is more concern for underexposure, leading to increased relapse, than for overexposure.^14^ Overexposure is of particular concern for patients at the lower end of each weight band, as it may increase the risk of adverse events. Therefore, alongside AUC_0-EOT_, we also compared simulated *C*_max,0-EOT_ values with those previously observed for the allometric FFM-based and mg/kg regimens (data not shown).^15,16,22^ Miltefosine keeps accumulating during treatment, and *C*_max_ is expected in the last week of treatment. All simulated *C*_max,0-EOT_ values were within the range of those previously observed (max 59.1 µg/mL). The main safety concern for miltefosine is mild-to-moderate gastrointestinal adverse events. These occur most often at the start of treatment, when systemic miltefosine plasma concentrations are still relatively low and are likely attributable to the direct activity of the drug’s amphiphilic surfactant properties on the gastrointestinal lining.^38^ The impact of the selected doses on target attainment is clearer within the individual kg weight bins of the FFM- and WB-based regimens (Figure S2), while the distribution of individual values still demonstrates good overlap.

The population PK model by Verrest *et al*.^21^ selected for this study was built on the largest number of only VL patients in Eastern Africa receiving the allometric FFM-based miltefosine regimen to date (*n* = 239). Various miltefosine PK models have been published for other clinical forms of leishmaniasis, including post-kala-azar dermal leishmaniasis (PKDL) and cutaneous leishmaniasis (CL).^39,40^ Exposure–response relationships and associated PK targets for these different clinical phenotypes have not been established yet, still we suggest that the same WB-based doses may be applicable, as allometric dosing has also been recommended for paediatric CL and PKDL patients.^40–42^ The PK exposure targets are sensitive to simulation settings such as sample size, age distribution and seed number. They were used solely to guide dose selection and should not be directly extrapolated to clinical practice without further validation. In particular, the upper AUC_0-EOT_ target is not based on an established relationship with observed toxicity.

Proposing a dosing regimen without direct validation through clinical trials carries inherent risks but is not unprecedented. For instance, the WHO has accepted simulation-based paediatric dosing recommendations for bedaquiline and delamanid for the treatment of drug-resistant tuberculosis, as well as paediatric dose optimization in rare diseases due to the limited number of available patients,^43,44^ and regulatory agencies such as the EMA explicitly recognize modelling as an alternative to clinical trials.^45^ By yielding a similar exposure profile as the existing FFM-based dosing, our WB-based regimen does not need to be validated through a clinical trial and can benefit from pharmacovigilance monitoring to certify its effectiveness. A key limitation of using geographically defined populations is the possible lack of generalizability to other regions, such as India, Nepal and Brazil, where VL is also endemic.^46,47^ A comparison of weight and height data from Indian paediatric VL patients (*n* = 814), kindly provided by DNDi, revealed similar median values and distribution of weight- and height-for-age curves compared with Eastern African patients with VL (Figure S3). Furthermore, the allometric FFM-based regimen, shown to be effective in Eastern Africa, was originally developed using clinical trial data from Indian patients.^17^ This suggests that the proposed WB-based regimen is suitable for the Indian subcontinent as well. For the Americas region, the proposed WB-based regimen is also expected to yield equivalent exposure in paediatrics compared with the currently recommended 2.5 mg/kg/day adult dose regimen, as outlined in the most recent Pan American Health Organization treatment guidelines; therefore, we suggest that it may also be used in this region.^48^

In conclusion, this study proposes a WB-based allometric miltefosine regimen with similar exposure and target attainment compared with the FFM-based allometric regimen, offering a more practical solution for paediatric VL treatment. This model-informed approach negates the need for extensive paediatric clinical trials.

## Supplementary Material

dkag014_Supplementary_Data

## Supplementary data

Figures S1–S3 and Tables S1–S3 are available as Supplementary data at *JAC* Online.
