# Supplementary material for: Weight-band-based simplification of oral allometric miltefosine dosing in paediatric patients with visceral leishmaniasis
Source: J Antimicrob Chemother. 2026 Jan 23;81(2):dkag014. doi: 10.1093/jac/dkag014 (PMC12828427; doi:10.1093/jac/dkag014)
Supplement: dkag014_Supplementary_Data [file dkag014_supplementary_data.docx]

**Supporting information**

**Table S1.** Daily allometric miltefosine dose for boys (upper) and girls (lower) based on FFM.

| Boys | | | | | | | | | | | |
| --- | --- | --- | --- | --- | --- | --- | --- | --- | --- | --- | --- |
| HT (cm) | 60-69 | 70-79 | 80-89 | 90-99 | 100-109 | 110-119 | 120-129 | 130-139 | 140-149 | 150-159 | 160-169 |
| WT (kg) |  |  |  |  |  |  |  |  |  |  |  |
| 4 | 20 | 20 |  |  |  |  |  |  |  |  |  |
| 5 | 20 | 30 |  |  |  |  |  |  |  |  |  |
| 6 | 30 | 30 | 30 |  |  |  |  |  |  |  |  |
| 7 | 30 | 30 | 30 |  |  |  |  |  |  |  |  |
| 8 | 30 | 30 | 40 | 40 | 40 | 40 | 40 |  |  |  |  |
| 9 | 30 | 40 | 40 | 40 | 40 | 40 | 40 |  |  |  |  |
| 10 | 40 | 40 | 40 | 40 | 40 | 50 | 50 |  |  |  |  |
| 11 | 40 | 40 | 40 | 40 | 50 | 50 | 50 |  |  |  |  |
| 12 | 40 | 40 | 40 | 50 | 50 | 50 | 50 | 50 |  |  |  |
| 13 | 40 | 40 | 50 | 50 | 50 | 50 | 60 | 60 | 60 |  |  |
| 14 | 40 | 40 | 50 | 50 | 50 | 60 | 60 | 60 | 60 |  |  |
| 15 | 40 | 40 | 50 | 50 | 60 | 60 | 60 | 60 | 60 |  |  |
| 16 | 40 | 50 | 50 | 50 | 60 | 60 | 60 | 60 | 70 |  |  |
| 17 | 40 | 50 | 50 | 60 | 60 | 60 | 60 | 70 | 70 |  |  |
| 18 | 40 | 50 | 50 | 60 | 60 | 60 | 70 | 70 | 70 |  |  |
| 19 | 40 | 50 | 50 | 60 | 60 | 70 | 70 | 70 | 70 |  |  |
| 20 | 40 | 50 | 50 | 60 | 60 | 70 | 70 | 70 | 70 | 80 |  |
| 21 | 40 | 50 | 60 | 60 | 60 | 70 | 70 | 70 | 80 | 80 |  |
| 22 | 40 | 50 | 60 | 60 | 70 | 70 | 70 | 80 | 80 | 80 | 80 |
| 23 | 40 | 50 | 60 | 60 | 70 | 70 | 80 | 80 | 80 | 80 | 80 |
| 24 | 40 | 50 | 60 | 60 | 70 | 70 | 80 | 80 | 80 | 80 | 100 |
| 25 | 50 | 50 | 60 | 60 | 70 | 70 | 80 | 80 | 80 | 100 | 100 |
| 26 | 50 | 50 | 60 | 70 | 70 | 80 | 80 | 80 | 100 | 100 | 100 |
| 27 | 50 | 50 | 60 | 70 | 70 | 80 | 80 | 80 | 100 | 100 | 100 |
| 28 | 50 | 50 | 60 | 70 | 70 | 80 | 80 | 100 | 100 | 100 | 100 |
| 29 | 50 | 50 | 60 | 70 | 70 | 80 | 80 | 100 | 100 | 100 | 100 |

kg = kilograms, cm = centimeters

| Girls | | | | | | | | | | | |
| --- | --- | --- | --- | --- | --- | --- | --- | --- | --- | --- | --- |
| HT (cm) | 60-69 | 70-79 | 80-89 | 90-99 | 100-109 | 110-119 | 120-129 | 130-139 | 140-149 | 150-159 | 160-169 |
| WT (kg) |  |  |  |  |  |  |  |  |  |  |  |
| 4 | 20 |  |  |  |  |  |  |  |  |  |  |
| 5 | 20 | 20 |  |  |  |  |  |  |  |  |  |
| 6 | 20 | 20 | 30 |  |  |  |  |  |  |  |  |
| 7 | 20 | 30 | 30 | 30 | 30 |  |  |  |  |  |  |
| 8 | 30 | 30 | 30 | 30 | 30 |  |  |  |  |  |  |
| 9 | 30 | 30 | 30 | 30 | 30 |  |  |  |  |  |  |
| 10 | 30 | 30 | 30 | 40 | 40 | 40 | 40 |  |  |  |  |
| 11 | 30 | 30 | 40 | 40 | 40 | 40 | 40 |  |  |  |  |
| 12 | 30 | 30 | 40 | 40 | 40 | 40 | 40 |  |  |  |  |
| 13 | 30 | 40 | 40 | 40 | 40 | 40 | 50 |  |  |  |  |
| 14 | 30 | 40 | 40 | 40 | 40 | 50 | 50 | 50 | 50 |  |  |
| 15 | 30 | 40 | 40 | 40 | 50 | 50 | 50 | 50 | 50 |  |  |
| 16 | 30 | 40 | 40 | 50 | 50 | 50 | 50 | 50 | 50 |  |  |
| 17 | 40 | 40 | 40 | 50 | 50 | 50 | 50 | 60 | 60 |  |  |
| 18 | 40 | 40 | 50 | 50 | 50 | 50 | 60 | 60 | 60 |  |  |
| 19 | 40 | 40 | 50 | 50 | 50 | 60 | 60 | 60 | 60 |  |  |
| 20 | 40 | 40 | 50 | 50 | 50 | 60 | 60 | 60 | 60 | 60 | 60 |
| 21 | 40 | 40 | 50 | 50 | 60 | 60 | 60 | 60 | 60 | 70 | 70 |
| 22 | 40 | 40 | 50 | 50 | 60 | 60 | 60 | 60 | 70 | 70 | 70 |
| 23 | 40 | 40 | 50 | 50 | 60 | 60 | 60 | 70 | 70 | 70 | 70 |
| 24 | 40 | 50 | 50 | 50 | 60 | 60 | 60 | 70 | 70 | 70 | 70 |
| 25 | 40 | 50 | 50 | 60 | 60 | 60 | 70 | 70 | 70 | 70 | 70 |
| 26 | 40 | 50 | 50 | 60 | 60 | 60 | 70 | 70 | 70 | 70 | 80 |
| 27 | 40 | 50 | 50 | 60 | 60 | 70 | 70 | 70 | 70 | 80 | 80 |
| 28 | 40 | 50 | 50 | 60 | 60 | 70 | 70 | 70 | 80 | 80 | 80 |
| 29 | 40 | 50 | 50 | 60 | 60 | 70 | 70 | 70 | 80 | 80 | 80 |

kg = kilograms, cm = centimeters

**Table S2.** Population PK parameter estimates of the previously published miltefosine pharmacokinetic model by Verrest *et al.*

|  |  | Estimate | 95% CI^F^ |
| --- | --- | --- | --- |
| Structural parameters | |  |  |
|  | CL (L/day)^A^ | 1.85 | 1.75-1.94 |
|  | Vc (L)^B^ | 13.6 | 12.8-14.4 |
|  | Q (L/day) | 0.17 | 0.13-0.21 |
|  | Vp (L) | 2.22 | 1.96-2.59 |
|  | k_a_ (day^-1^) | 0.037 | 0.036-0.038 |
|  | F^C^ | 1 (fixed) |  |
|  | COV_F,W1_ (fractional change)^D^ | -0.64 | -0.57 to -0.73 |
|  | COV_F,CD_ (exponent)^E^ | -2.40 | -3.79 to -1.21 |
| Between-subject variability | |  |  |
|  | CL (CV%) | 16.3 | 14.3-18.5 |
|  | COV_F,W1_ (CV%) | 74.8 | 62.0-90.3 |
| Residual unexplained variability | |  |  |
|  | Proportional error (CV%) | 31.5 | 29.7-33.6 |

^A^ Typical value of CL (CL_TV_) is allometrically scaled (${Cl}_{TV}={Cl}_{pop}\times{({{FFM}_{i}}/{{FFM}_{median}})}^{0.75}$) where FFM_med_ = 18 kg

^B^ Typical value of Vc (Vc_TV_) is allometrically scaled (${Vc}_{TV}={Vc}_{pop}\times{({{FFM}_{i}}/{{FFM}_{median}})}^{1.00}$) where FFM_med_ = 18 kg

^C^ Typical value of F (F_TV_) has two covariates ($F_{TV}= F_{pop}\times(1+ {COV}_{F,W1})\times{({{CD}_{i,t}}/{{CD}_{median}})}^{{COV}_{F,CD}})$

^D^ Fractional change in oral bioavailability during the first week of treatment.

^E^ Effect of the cumulative dose (CD) in the oral bioavailability upon reaching a certain threshold (60 mg/kg)

^F^ Confidence intervals obtained by sampling importance resampling (SIR).

**Table S3.** Correction factor functions for scaling height and weight per sex.

| Metric | Sex | Equation |
| --- | --- | --- |
| Height (cm) | Boy | $CF={-7.60E}^{-2}HT+{2.39E}^{-2}{HT}^{2}-{3.00E}^{-3}{HT}^{3}+ {1.62E}^{-4}{HT}^{4}- {3.17E}^{-6}{HT}^{5}+1.06$ |
|  | Girl | $CF={-7.53E}^{-2}HT+{2.69E}^{-2}{HT}^{2}-{3.67E}^{-3}{HT}^{3}+ {2.14E}^{-4}{HT}^{4}- {4.44E}^{-6}{HT}^{5}+1.04$ |
| Weight (kg) | Boy | $CF={-1.06E}^{-1}WT+{3.77E}^{-2}{WT}^{2}-{5.16E}^{-3}{WT}^{3}+ {2.88E}^{-4}{WT}^{4}- {5.63E}^{-6}{WT}^{5}+0.99$ |
|  | Girl | $CF={-6.36E}^{-2}WT+{2.26E}^{-2}{WT}^{2}-{3.34E}^{-3}{WT}^{3}+ {2.01E}^{-4}{WT}^{4}- {4.55E}^{-6}{WT}^{5}+0.86$ |

The functions presented were obtained by comparing the value of height and weight per age-bin (age rounded to the nearest integer) of the available demographic VL Eastern Africa pediatric population (n = 9,379) to the WHO-CDC growth curves-generated children (n = 130,200). The ratio of the median weight/height of the demographic VL patients’ values to a median of WHO-CDC generated population values was plotted against the age-bin, and a 5-degree polynomial regression was fitted per metric, per sex. To obtain the transformed weight or height the value has to be multiplied by the corresponding CF. CF: correction factor; WT: weight; HT: height; cm: centimeters; kg: kilograms.


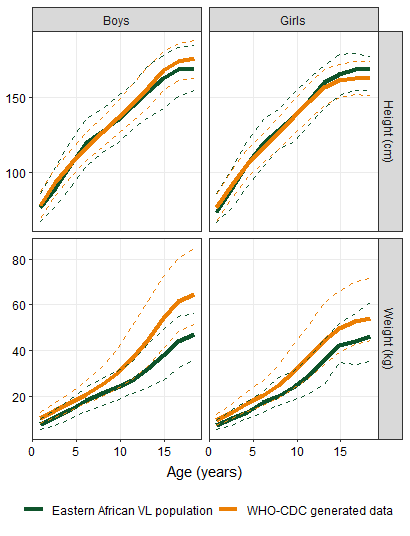


**Figure S1**. Stratified sex distribution of height (upper panels) and weight (bottom panels) for boys (left column) and girls (right column). A summary of the observed Eastern African VL population (green) and the non-adjusted combined WHO-CDC data (orange) is shown in all panels with the median (solid line) and 5^th^ and 95^th^ percentiles (dashed lines) in their respective colors.


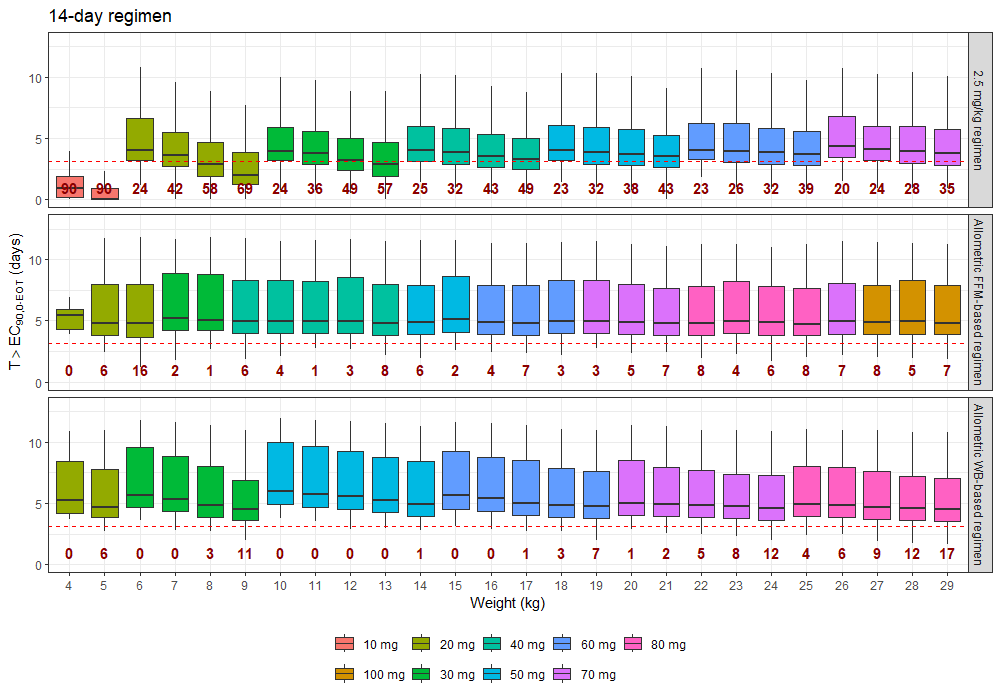

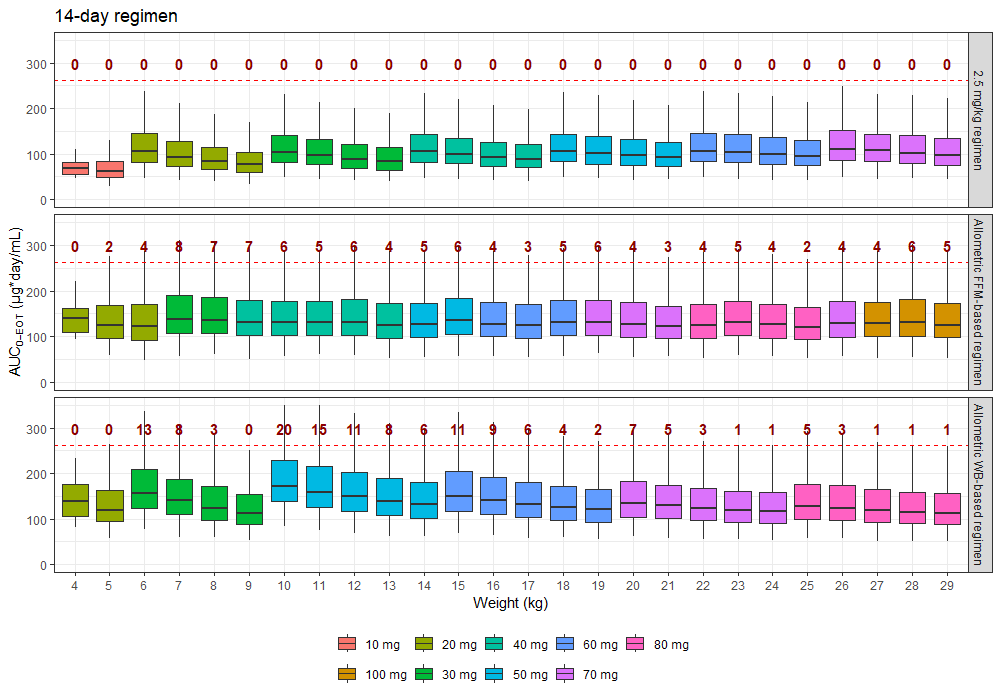

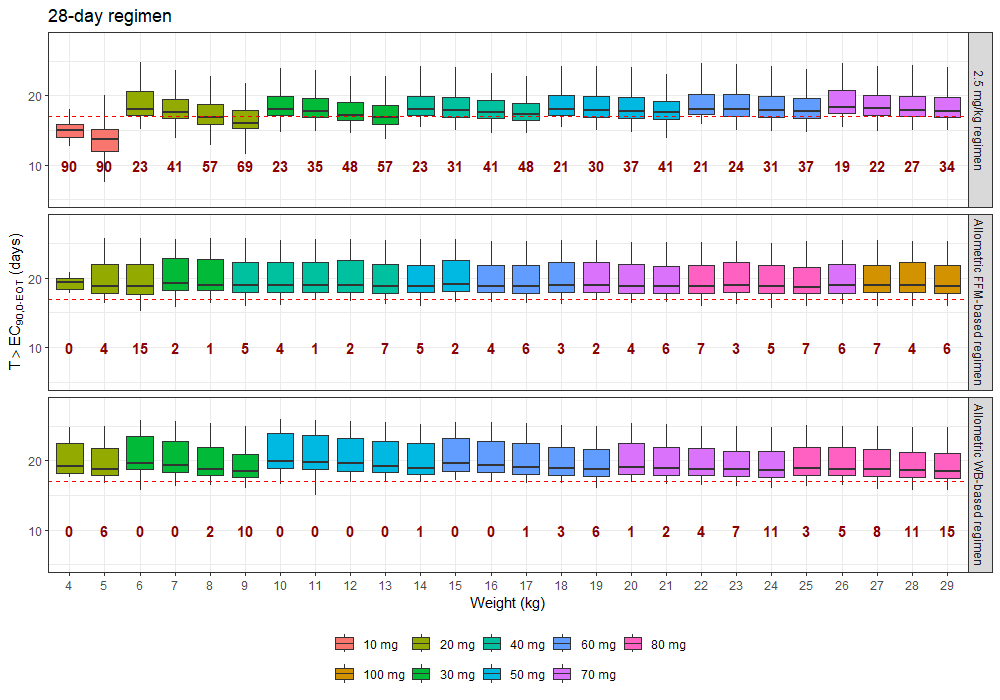


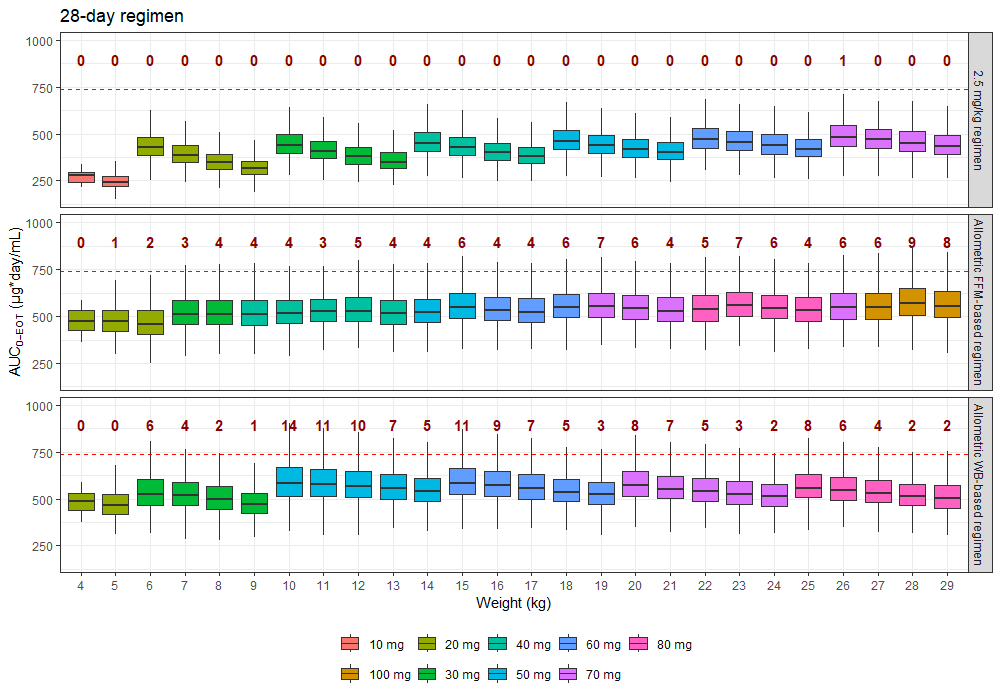


**Figure S2**. Distribution of the target PK metrics of the three dosing regimens evaluated. PK target distribution of per kg is presented in individual boxplots. The number in red is the percentage of individuals in each weight-bin that are over or below the target exposure. The horizontal dashed line is the selected target for each duration. Boxplots are colored according to the mode of doses in the case of the 2.5 mg/kg and the FFM-based regimen, whilst for the WB-based regimen the selected dose is presented for each weight bin in every dosing regimen.


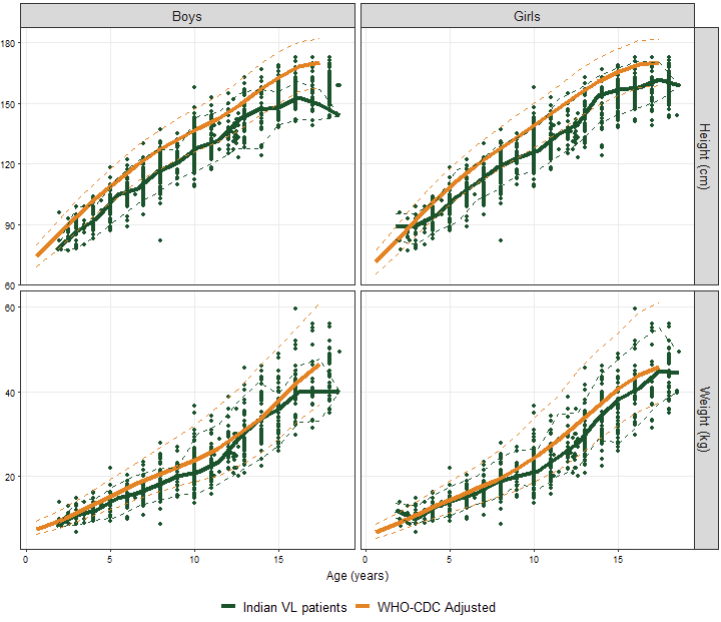


**Figure S3**. Stratified sex distribution of height (upper) and weight (lower) with the inclusion of pediatric VL patients from India (n = 814) in the green dots. The continuous lines represent the mean value of the metric with the corresponding 95^th^ and 5^th^ percentiles in dashed lines. The available data was provided by the Drugs for Neglected Diseases initiative, from patients with a confirmed VL diagnosis.
